# Adverse events reporting of XPO1 inhibitor - selinexor: a real-word analysis from FAERS database

**DOI:** 10.1038/s41598-024-62852-z

**Published:** 2024-05-28

**Authors:** Yi Liu, Runyu Yang, Hui Feng, Yue Du, Bingyu Yang, Mengyao Zhang, Pengcheng He, Bohan Ma, Fan Niu

**Affiliations:** 1 Department of Hematology, The First Affiliated Hospital of Xi’an Jiaotong University, No. 277 Yanta West Road, Xi’an, 710061 Shaanxi China; 2 Institute of Medical Research, Northwestern Polytechnical University, No.127 Friendship West Road, Beilin District, Xi’an, 710072 Shaanxi China

**Keywords:** Selinexor, FAERS, Adverse events, Drug safety, Diseases, Medical research

## Abstract

As the world's first oral nuclear export inhibitor, selinexor is increasingly being used in clinical applications for malignant tumors. However, there is no extensive exploration on selinexor's adverse events (ADEs), necessitating a real-word assessment of its clinical medication safety. FAERS data (July 2019–June 2023) were searched for selinexor ADE reports across all indications. Use the system organ class (SOC) and preferred terms (PT) from the medical dictionary for regulatory activities (MedDRA) to describe, categorize, and statistic ADEs. Disproportionality analysis was employed through calculation of reporting odds ratio (ROR) and proportional reporting ratio (PRR). Based on total of 4392 selinexor related ADE reports as the primary suspect (PS), of which 2595 instances were severe outcomes. The predominant ADEs included gastrointestinal disorders, myelosuppression symptoms, and various nonspecific manifestations. 124 signals associated with selinexor ADE were detected, and 10 of these top 15 signals were not included into the instructions. Our study provides real-world evidence regarding the drug safety of selinexor, which is crucial for clinicians to safeguard patients’ health.

## Introduction

Selinexor is a selective inhibitor of nuclear export (SINE) primarily targeting XPO1 protein^1,2^. As the first orally administered nuclear export inhibitor around the world, selinexor exerts its anticancer properties by blocking the export of tumor suppressor proteins (TSPs), such as p53, FOXO, RB, etc., from the cell nucleus to cytoplasm^3^. This behavior maximizes the tumor-suppressing effects of these proteins, which leads to the activation of downstream signaling pathways, ultimately resulting in apoptosis and death of cancer cells. Based on the results of phase 3 clinical trial (BOSTON)^4^, selinexor was approved by the FDA in July 2019 for use in relapsed or refractory multiple myeloma (RRMM)^5^. Subsequently, it was further approved for treatment in patients with relapsed or refractory diffuse large B-cell lymphoma (RR-DLBCL) who have received at least two prior therapies, bringing hope for the treatment of hematologic malignancies^6,7^. As a novel small molecule compound, selinexor has also generated significant interest in the oncology field, offering a potential option for treating various cancers^8^ with high XPO1 expression. Currently, hundreds of clinical trials are undergoing to further expand the indications of selinexor, including lung cancer^9,10^, prostate cancer^11,12^, endometrial cancer^13,14^, glioblastoma^15^, and other solid tumors^8,16,17^, etc.

Just like other pharmaceutical intervention, assessment of selinexor’s safety profile is imperative to ensure patients well-being. Despite its wide therapeutic promise, selinexor’s use has been associated with a range of adverse events (ADEs). Researches on selinexor ADEs indicated that the most common was myelosuppression^18^, followed by gastrointestinal disorders^19,20^, and nonspecific side effects^19,21^. Most descriptions of its ADEs previously rely on clinical trial observations, lacking certain studies based on real-world analysis. Moreover, strict clinical trial inclusion criteria and limited sample size may not fully predict the drug's effects and safety in a broader and more diverse population. Therefore, ADE reports associated with selinexor in actual might extend beyond the previously documented symptoms. In this context, there is an urgent need for more specific researches to assess the safety profiles related to selinexor.

FDA Adverse Event Reporting System (FAERS) is a database for collecting and monitoring adverse reactions on drugs and vaccines, aiding the FDA in assessing the risk–benefit balance of them^22^, thereby safeguarding public health. Numerous drugs for clinical use have undergone specific analysis via the FAERS database^23–25^, and make majority of patients benefit a lot. The real-world data provided by FAERS can reflect the wider population's experience with medication, offering insights beyond the controlled environment of clinical trials. Additionally, due to the long-term onset, these ADEs that may not be captured in clinical trials could also be identified through prolonged monitoring processes. Consequently, continuous monitoring through FAERS is a key component of post-marketing surveillance, ensuring ongoing evaluation of selinexor's safety as its use in clinical practice evolves.

In summary, selinexor offers a promising therapeutic choice for specific malignancies. Nevertheless, the specific evaluation of its safety use is required. This study aims to investigate selinexor's post-market ADEs through FAERS database, contributing to the evidence of safety profile in clinical.

## Methods

### Data extraction

All data in this study were sourced from selinexor-related ADE reports collected in the FAERS database. Raw data files were downloaded directly from the FDA official website (https://www.fda.gov). The data extracted for this study span from July 2019 (approved for marketing) to June 2023. The search terms used were “selinexor” and “XPOVIO”. The FAERS database is composed of seven distinct data files, which include information on demographic and administrative (DEMO), drug details (DRUG), reported adverse events (REAC), outcomes for patients (OUTC), sources of reports (RPSR), drug therapy (THER), and indications for drug use (INDI).

### Records screened

According to the FDA recommended deduplication reporting method, select the PRIMARY_ID, CASE_ID, and FDA_DT fields of the DEMO table, and sort them in the order of CASE_ID, FDA_DT, and PRIMARY_ID. For reports with the same CASE_ID, keep the one with the largest FDA_DT value. Furtherly, for reports with the same CASE_ID and FDA_DT, keep the one with the largest PRIMARY_ID value.

### Data processing

**Figure 1:**
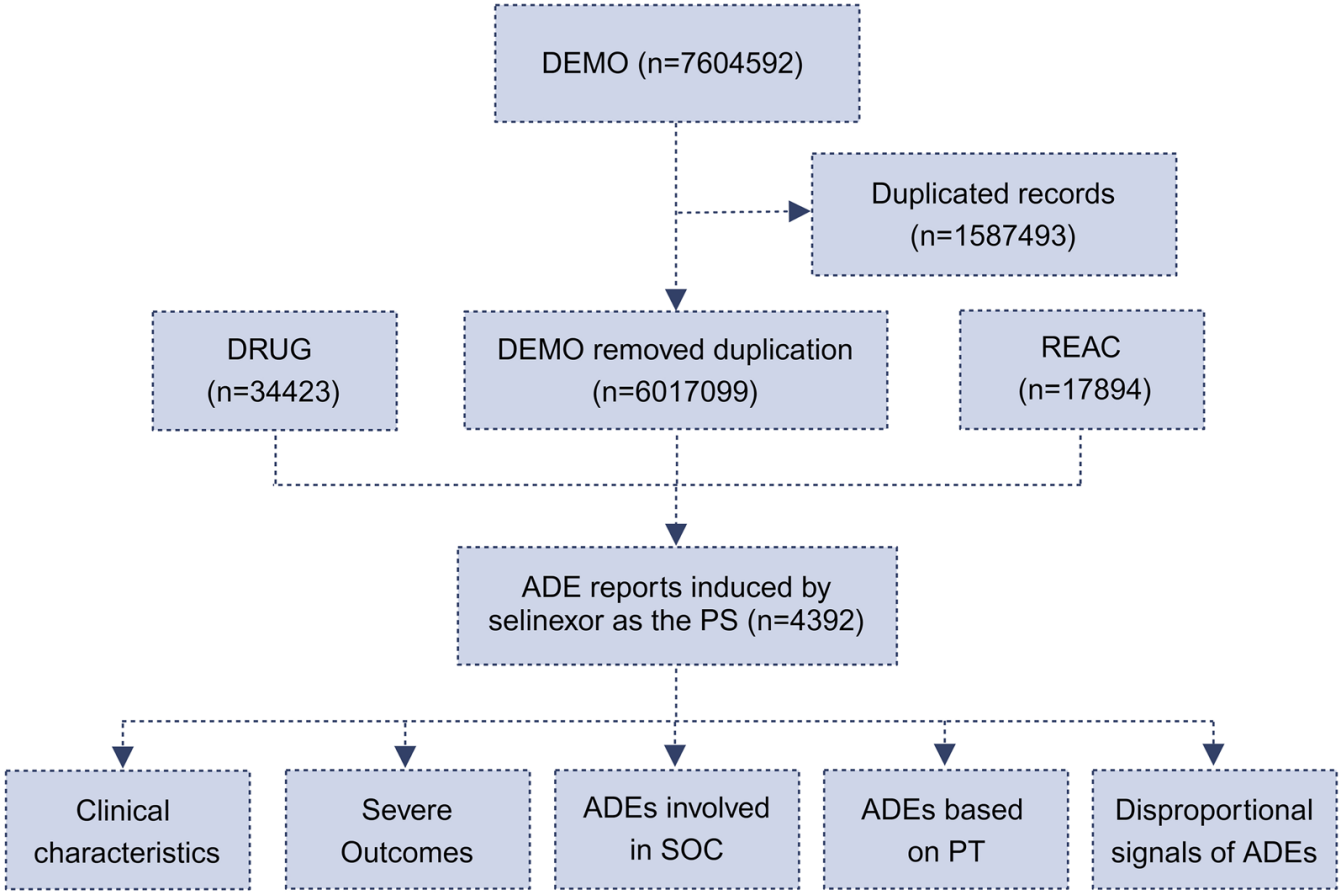
Flow chart of the study.

In DEMO data, it documented 7,604,592 of ADE reports associated with selinexor. After excluding duplicates records (n = 1,587,493), the final DEMO was determined (n = 6,017,099). After filtering through the DRUG (n = 34,423), we identified these reports by considering selinexor as the primary suspect drug (PS), while excluding data of secondary suspect (SS) and concomitant (C) drug. Together with REAC (n = 17,894), a total of 4392 selinexor related ADE reports as the PS were ultimately confirmed. The system organ class (SOC) and preferred term (PT) based on the medical dictionary for regulatory activities (MedDRA) facilitated the description, code and classification of the ADEs. Subsequent analysis was conducted as shown in Fig. 1.

### Statistical analysis

The reporting odds ratio (ROR) and proportional reporting ratio (PRR) methods were employed in this study, through a disproportional approach^26–28^. These two methods primarily rely on a four-cell table and calculate the ROR and PRR values using a corresponding formula. A higher numerical value indicates a stronger signal, signifying a stronger association between the target drug and the specific ADE. The calculation formulas and thresholds are presented in Table 1. SAS 9.4 software and GraphPad 8.4 software were employed for database analysis and results statistics.

**Table 1:** Calculation formulas and corresponding thresholds for ROR and PRR methods.

Methods	Formulas	Thresholds
ROR	${\text{ROR}} = \, ({\text{a}}/{\text{c}}) \, /({\text{b}}/{\text{d}})$ $$\begin{gathered} {\text{ROR 95}}\% \;{\text{CI }} = {\text{ e}}^{{{\text{[In }}({\text{ROR}}) \pm {1}.{96}\sqrt {\frac{1}{a} + \frac{1}{b} + \frac{1}{c} + \frac{1}{d}} ]}} \hfill \\ \hfill \\ \end{gathered}$$	a ≥ 3, Lower limit value of ROR 95% CI > 1, it indicates the generation of one signal
PRR	${\text{PRR}} = \, [{\text{a }}/({\text{a}} + {\text{c}}) \, ]/[{\text{b }}/({\text{b}} + {\text{d}}) \, ]$ $\chi^{{2}} = ({\text{ad}} - {\text{bc}})^{{2}} ({\text{a}} + {\text{b}} + {\text{c}} + {\text{d}})/ \, [({\text{a}} + {\text{b}})({\text{c}} + {\text{d}})({\text{a}} + {\text{c}})({\text{b}} + {\text{d}}) \, ]$	a ≥ 3, PRR > 2, *χ*^2^ > 4, it indicates the generation of one signal

a, number of target event reports of target drugs; b, other event reports of the target drug; c, target event reports of other drugs; d, other event reports of other drugs; 95% CI, 95% confidence interval.

## Results

### Annual distribution of selinexor ADE reports

**Figure 2:**
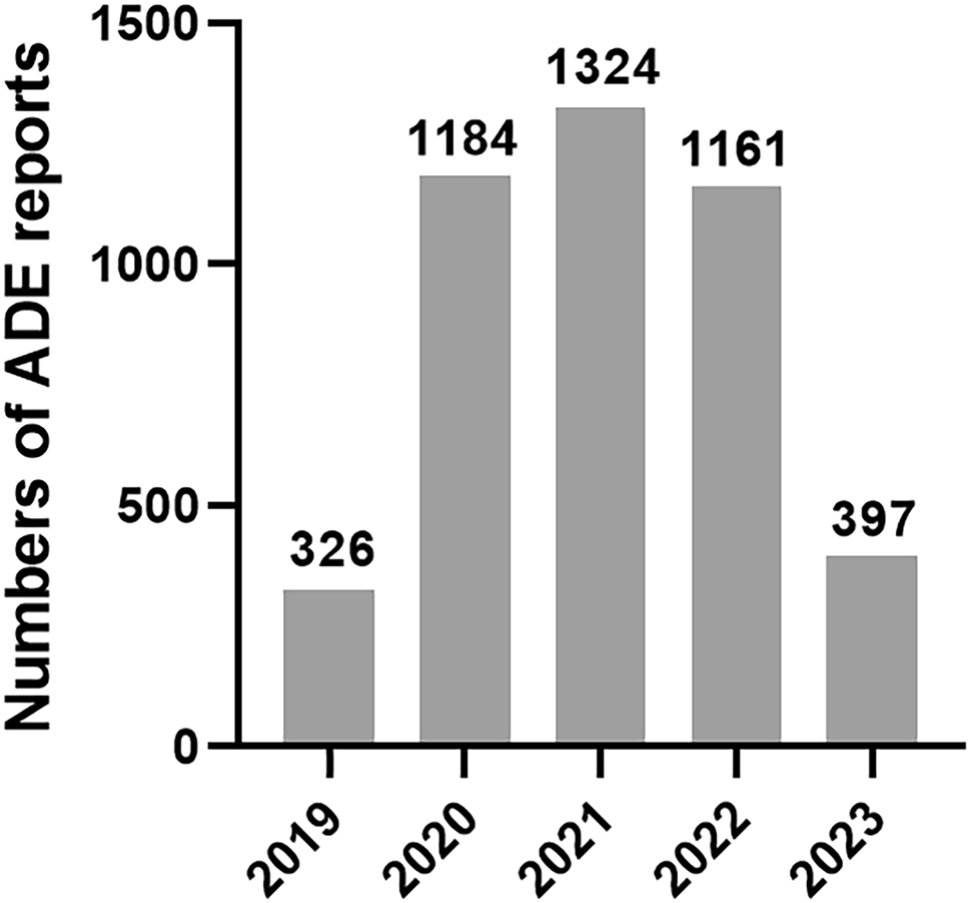
Annual distribution of selinexor ADE reports.

From July 2019 to June 2023, the FDA received a total of 4392 selinexor related ADE reports as the PS. Statistical results indicate an overall upward trend in the number of reports, with specific distributions demonstrated in Fig. 2.

### Clinical characteristics distribution of selinexor ADE reports

There were 4392 reports, with detailed distributions of basic clinical characteristics presented in Table 2. The results indicate that a significant portion of patient information was unknown (gender unreported for 69.7%, age unreported for 98%, weight unreported for 87.1%, respectively). Regarding other aspects, the statistics on reporter identification indicate that 62.9% of the reports came from consumers or patients themselves, followed by physicians (22.0%), healthcare professionals (12.0%), pharmacists (1.0%), and non-healthcare professionals (0.8%). Additionally, these received selinexor ADE reports among the countries and regions worldwide, the top five reporting countries were the United States (74.8%), followed by China (4.0%), Canada (3.1%), Israel (2.7%), and Australia (2.6%), respectively (Table 2).

**Table 2:** Clinical characteristics of reports with selinexor ADEs.

Characteristics	Classification	Number (n)	Percentage (%)
Gender	Male	740	16.8
	Female	589	13.4
	N/A	3063	69.7
Age (year)	18–64	32	0.7
	≥ 65	54	1.2
	N/A	4306	98
Weight (kg)	< 80	371	8.4
	80–100	135	3.1
	> 100	62	1.4
	N/A	3824	87.1
Reported person	Consumer	2761	62.9
	Physician	968	22.0
	Health-profession	528	12.0
	Pharmacist	45	1.0
	Other health-profession	36	0.8
	Unknown	54	1.2
Reported countries (Top 5)	United States	3287	74.8
	China	176	4.0
	Canada	138	3.1
	Israel	118	2.7
	Australia	115	2.6

*N/A* not recorded information.

### Severe outcomes in selinexor

Among the 4392 selinexor related ADE reports overall, there were 2595 instances of severe outcomes, including hospitalization (55.3%), death (28.9%), life-threatening conditions (2.3%), disability (0.8%), permanent damage (n = 1), and others (12.6%). See Table 3 for details.

**Table 3:** The distribution of selinexor severe outcomes.

Severe outcomes	Reports	Percentage (%)
Hospitalized	1436	55.3
Death	750	28.9
Life-threatening	60	2.3
Disability	20	0.8
Resulted in permanent impairment/disability	1	0.0
Other	328	12.6

### Selinexor ADEs involved in SOC

**Figure 3:**
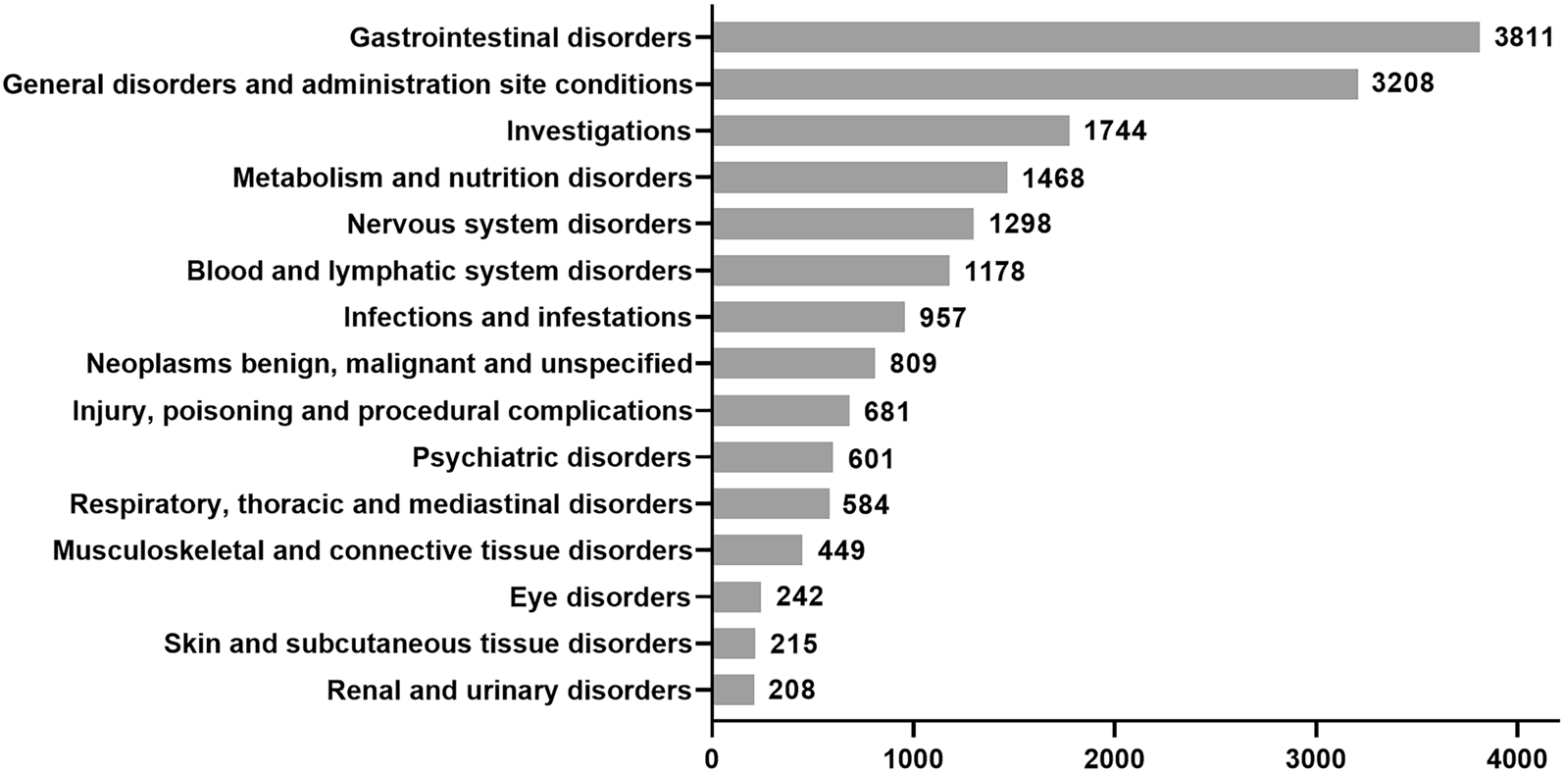
TOP15 SOCs involved in selinexor ADEs.

Out of the submitted 4392 reports, a total of 17,483 ADE instances were identified based on different SOC categories. There were predominantly gastrointestinal reactions (n = 3811, 21.80%), followed by general disorders and administration site conditions (n = 3208, 18.35%), investigations (n = 1744, 19.98%) meaning examination relevant abnormalities including various blood tests, biochemical analyses, imaging examinations, etc., as illustrated in Fig. 3.

### Top 15 ADEs Based on PT

Among the 4392 reports, there are 17,894 ADE instances occurrence. We compiled the top 15 ADEs based on PT description, primarily involving gastrointestinal disorders (nausea, anorexia or loss of appetite, diarrhea, vomiting, constipation) for 23.07% (n = 4128), hematologic abnormal investigations (thrombocytopenia, decreased platelet count, anemia) for 6.87% (n = 1229), and other non-specific symptoms (fatigue, lethargy, weight loss, dizziness) for 12.89% (n = 2307), etc. Detailed information is provided in Table 4.

**Table 4:** Top 15 selinexor reported ADEs.

NO	PT	Number of reports	Percentage (%)
1	Nausea	1568	8.76
2	Fatigue	1151	6.43
3	Anorexia or loss of appetite	919	5.14
4	Diarrhea	774	4.33
5	Vomiting	605	3.38
6	Thrombocytopenia	525	2.93
7	Death	466	2.60
8	Lethargy	463	2.59
9	Weight Loss	432	2.41
10	Decreased platelet count	399	2.23
11	Anemia	305	1.70
12	Constipation	262	1.46
13	Dizziness	261	1.46
14	Infectious pneumonia	233	1.30
15	Dehydration	203	1.13

### Top ADE signals based on PT

In the analysis of 4392 selinexor-related ADE reports identified as the PS, using both PRR and ROR screening methods, we identified 124 ADE signals. Notably, 10 of the top 15 signals were neither listed in the prescribing information nor reported in the literature. These signals were illustrated as neurofibrosarcoma (ROR, 144.27; 95% Cl, 61.16–340.32), hypercreatinemia (ROR, 113.92; 95% CI, 57.08–227.37), refractory diffuse large B-cell lymphoma (ROR, 107.67; 95% CI, 77.63–149.33), hypercreatininemia (ROR, 74.00; 95% CI, 37.56–145.79), meibomial gland dysfunction (ROR, 37.57; 95% CI, 15.37–91.81), sarcoma (ROR, 28.20; 95% CI, 14.53–54.73), hypogeusia (ROR, 20.69; 95% CI, 11.06–38.71), abnormal kidney function (ROR, 13.75; 95% CI, 8.74–21.63), bone tumors (ROR, 13.08; 95% CI, 4.88–35.10), iridesma (ROR, 11.31; 95% CI, 3.62–35.32), etc. (Table 5).

**Table 5:** Top 15 disproportional signals for ADEs associated with selinexor.

NO	PT	Number of reports	PRR	ROR (95% Cl)
1	Neurofibrosarcoma^b^	6	144.222393	144.27 (61.16–340.32)
2	Hypercreatinemia^a^	9	113.8597839	113.92 (57.08–227.37)
3	Refractory DLBCL^b^	40	107.4282257	107.67 (77.63–149.33)
4	Hypercreatininemia^a^	9	73.96020153	74.00 (37.56–145.79)
5	Meibomian gland dysfunction^a^	5	37.55791484	37.57 (15.37–91.81)
6	Sarcoma^b^	9	28.1867869	28.20 (14.53–54.73)
7	Hypogeusia^a^	10	20.67704559	20.69 (11.06–38.71)
8	Thrombocytopenia	525	18.08658051	18.60 (17.04–20.31)
9	Pancytopenia	92	14.57752159	14.65 (11.92–18.01)
10	Anorexia or loss of appetite	919	14.19396208	14.91 (13.94–15.94)
11	Abnormal renal function^a^	19	13.735466	13.75 (8.74–21.63)
12	Bone tumors^b^	4	13.08139619	13.08 (4.88–35.10)
13	Decreased platelet count	399	12.44385369	12.70 (11.50–14.04)
14	Influenza-like Pneumonia	4	11.37849254	11.38 (4.25–30.50)
15	Iridesma^a^	3	11.31156023	11.31 (3.62–35.32)

^a^Indicates the ADEs which were not included in the instruction manual.

^b^Indicates that selinexor has undergoing clinical trials for this disease, meaning a potential indication.

## Discussion

This study utilized the FAERS database to rigorously evaluate ADEs associated with selinexor's clinical use. Notably, we observed an elevated occurrence of events leading to severe outcomes that could not be ignored. This requires the focus of patient health, regulatory authorities as well as pharmaceutical companies. Based on PT description, major ADEs identified included myelosuppression, gastrointestinal disorders, and non-specific general reactions in our study. Regarding as ADEs involved in SOC, given that the administration manner of oral drugs, the top ADEs were generally refer to be gastrointestinal tract (GI), aligning with the premarketing evaluations. However, some inconsistent and unreported ADE signals emerged compared to previous reports, which deserve more attention.

Hypercreatinemia, typically defined as elevated blood creatine level, is usually associated with issues of musculoskeletal, connective tissue, and metabolism. It has not been explicitly reported in public studies on selinexor, indicating that such an ADE should be closely monitored in following clinical practice. Similar with hypercreatininemia, which is a sign of renal function abnormality, both of them reflect an imbalance of substances related to body metabolism. Yet, the causes for themselves are generally diverse. In FAERS analysis, hypercreatinemia and hypercreatininemia are categorized under varied SOC classes, thus the captured ADE signals differed. Regarding as issue of renal dysfunction, it was notably evidenced by 19 instances of abnormal renal function and 9 instances of hypercreatininemia in our results. It’s well-known that injury of renal may be caused by various factors including disease progression, infections, and the use of other certain medications, such as nonsteroidal anti-inflammatory drugs (NSAIDs)^29^, statins/fibrates lipid-lowering drugs^30^, certain antibiotics (aminoglycosides)^31^, antifungal agents (amphotericin B)^32^, and antipsychotics (olanzapine)^33^, etc. Nevertheless, our study focused on statistical analysis of ADEs which were primarily suspected to be related to selinexor, there was no need to consider of concomitant medication. We attempted to explore whether the use of selinexor is associated with renal impairment or not, and found there were only minority of studies elucidated previously^34^. A clinical trial on RR-DLBCL patients found that selinexor's safety and efficacy to be independent of renal function. The *post-hoc* analysis of the SADAL study indicates that selinexor remained safe and effective even in patients with impaired renal function^35^. In assessments of MM, *Bader’s* study suggested the use of selinexor cannot aggravate the side effects on renal injuries^34^, regardless of the patient's prior renal function. Furthermore, descriptions of renal function impairment in ADE signals involve many abnormal indicators, including electrolyte level abnormalities. For instance, hyponatremia occurrences were reported frequently in previous studies, with an incidence rate about 30%^36^. In our study, we did not find specific signals linked selinexor ADEs to electrolyte imbalance. Taken together, the direct association of selinexor with renal function abnormality appears insufficient in clinical applications. We're not sure if it is caused by other concomitant medications, and look forward to further studies in the future.

Regarding issues on tumor signals following the clinical application of selinexor, we could not conclude a definite causal relationship. As shown in the results, our study showed that several ADE signals with tumors were involved, such as neurofibrosarcoma, refractory DLBCL, sarcoma, and bone tumors, etc. Because our analysis did not set a specific application scope, it suggests that patients for potential indications might have experienced progression of their own diseases, due to selinexor is currently under clinical trials for numerous hematologic malignancies and solid tumors. For example, Gounder's et al.^37^ study assessed the pharmacokinetics, pharmacodynamics, safety, and efficacy of selinexor in patients with advanced soft tissue or bone sarcomas. Another study evaluated the safety of selinexor in patients with relapsed or refractory non-Hodgkin lymphoma (NHL), including subtypes of NHL such as diffuse large B-cell lymphoma, Richter transformation, mantle cell lymphoma, follicular lymphoma, and chronic lymphocytic leukemia^38^. Additionally, *Lewin’s* study explored the safety and efficacy of selinexor in combination with doxorubicin in treating advanced soft tissue sarcomas (STS), compassing malignant peripheral nerve sheath tumors (n = 3) and other types of sarcomas (n = 16)^39^. According to these results, selinexor has shown certain effectiveness in treating various solid tumors as a potential indication. Nevertheless, even if selinexor is highly associated with specific tumor signals, it is difficult to conclude that the drug directly caused the event.

Moreover, selinexor may cause a variety of non-specific side effects as a novel targeting anti-tumor drug. Nagham et al.^40^ retrospectively analyzed the medical records of 174 patients who received at least one dose of selinexor in combination with chemotherapy or immunotherapy drugs. This cohort monitored the occurrence of the ocular ADEs, with blurred vision (12.64%), followed by dry eye syndrome (12.1%), and progression of cataracts (4.0%). However, there were no discontinuations due to ocular ADEs, leading to the conclusion that selinexor-related ocular ADEs are mild. In our study, meibomian gland dysfunction and iridesma were reported for the first time, suggesting that we can offer distinct insights into selinexor related ADE signals. Another related ADE of taste reduction, the possible physiological mechanisms behind this side effect include the drug's direct toxic effect on taste cells or indirect impacts on the oral environment. Nevertheless, this non-specific ADE of selinexor is rarely reported in clinical, possibly because it is temporary which did not cause enough attention.

The discovery of these potential ADEs underscores the need for a deeper investigation into selinexor's safety profile. Firstly, the identification of these unreported ADEs highlights the importance of continuous drug monitoring post-marketing. Secondly, these findings remind us that with the expanding indications and broaden applications in real-world of selinexor, unexpected ADEs might emerge. Although clinical trials provide crucial data on safety and efficacy, they are usually employed in a strictly controlled environment with limited sample sizes, which may not fully represent a broader and more diverse patient population. By recognizing and understanding these new ADEs, interventions can be implemented to prevent the effects, leading to better patient outcomes and life quality.

However, the FAERS analysis also has its certain limitations. Self-reporting bias, the lack of controlled studies, and the complexity of determining causal relationships are inherent flaws in FAERS analysis^41^. As a spontaneous reporting system, it means the reports come from numerous sources, and over- or under-reporting of specific information may lead to reporting bias. Therefore, the quality, completeness and accuracy of the reports may be limited. For instance, “N/A” in the chart of our study signifies missing data from the up-loader. This lack of basic information may compromise data integrity, but the generation of ADE signals would not be affected due to the massive real-word data. Besides, ADE reports in FAERS could not establish a causal relationship between drug use and ADEs. In FAERS report, there was no control group to compare ADEs between drug users and non-users. Without a control group, it is difficult to determine whether ADEs caused by specific drug or other factors. Therefore, ADE reports in the FAERS should be interpreted cautiously. Although spontaneous report system provides an inadequate level of evidence hierarchy, it serves as a tool for timely and early assessment of safety issues, especially in new compounds. It's well-known that clinical trials are considered the best source of evidence, but they are often limited by stringent criteria and limited sample sizes. Conversely, the analysis of spontaneous reports reflects these conditions where patients experience primary outcomes within a complex real-world context.

In conclusion, our study offers the first real-world insights into oncological ADEs of selinexor, supplementing its post-marketing safety research and providing reference for its clinical use.

## Data Availability

Database is available on the FAERS official website. The raw/processed data reproduced these findings can be shared necessarily, which required the approval of the corresponding authors.
